# An exponential filter model predicts lightness illusions

**DOI:** 10.3389/fnhum.2015.00368

**Published:** 2015-06-24

**Authors:** Astrid Zeman, Kevin R. Brooks, Sennay Ghebreab

**Affiliations:** ^1^Department of Cognitive Science, ARC Centre of Excellence in Cognition and its Disorders, Macquarie UniversitySydney, NSW, Australia; ^2^Commonwealth Scientific and Industrial Research OrganisationMarsfield, NSW, Australia; ^3^Perception in Action Research Centre, Macquarie UniversitySydney, NSW, Australia; ^4^Department of Psychology, Macquarie UniversitySydney, NSW, Australia; ^5^Cognitive Neuroscience Group, Department of Psychology, University of AmsterdamAmsterdam, Netherlands; ^6^Intelligent Systems Lab Amsterdam, Institute of Informatics, University of AmsterdamAmsterdam, Netherlands

**Keywords:** exponential, filter, model, ODOG, lightness, illusion, contrast, assimilation

## Abstract

Lightness, or perceived reflectance of a surface, is influenced by surrounding context. This is demonstrated by the Simultaneous Contrast Illusion (SCI), where a gray patch is perceived lighter against a black background and vice versa. Conversely, assimilation is where the lightness of the target patch moves toward that of the bounding areas and can be demonstrated in White's effect. Blakeslee and McCourt (1999) introduced an oriented difference-of-Gaussian (ODOG) model that is able to account for both contrast and assimilation in a number of lightness illusions and that has been subsequently improved using localized normalization techniques. We introduce a model inspired by image statistics that is based on a family of exponential filters, with kernels spanning across multiple sizes and shapes. We include an optional second stage of normalization based on contrast gain control. Our model was tested on a well-known set of lightness illusions that have previously been used to evaluate ODOG and its variants, and model lightness values were compared with typical human data. We investigate whether predictive success depends on filters of a particular size or shape and whether pooling information across filters can improve performance. The best single filter correctly predicted the direction of lightness effects for 21 out of 27 illusions. Combining two filters together increased the best performance to 23, with asymptotic performance at 24 for an arbitrarily large combination of filter outputs. While normalization improved prediction magnitudes, it only slightly improved overall scores in direction predictions. The prediction performance of 24 out of 27 illusions equals that of the best performing ODOG variant, with greater parsimony. Our model shows that V1-style orientation-selectivity is not necessary to account for lightness illusions and that a low-level model based on image statistics is able to account for a wide range of both contrast and assimilation effects.

## 1. Introduction

**Figure 1:**
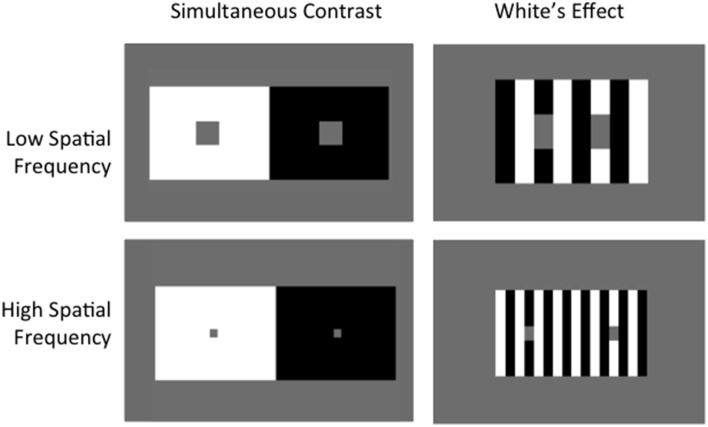
**Simultaneous Contrast vs. White's Effect**. Albedo of gray target patches in Simultaneous Contrast shift away from background, demonstrating contrast. Targets in White's Effect shift toward surrounding context, demonstrating assimilation. Increasing spatial frequency increases the effect in both cases.

Lightness is the perceived reflectance of a surface, which can vary greatly according to surrounding context, as demonstrated by lightness illusions (see Kingdom, 2011 for a recent review). One clear and well-known example is the Simultaneous Contrast Illusion (SCI), where a gray target patch is perceived as lighter when surrounded by a black background and darker when surrounded by a white background (Chevreul, 1839) (Figure 1 left). The SCI demonstrates the contrast phenomenon, where lightness shifts away from surrounding luminance values, luminance being the amount of light that reaches the eye. Under other circumstances, lightness can shift toward the luminance values of bordering areas—a phenomenon known as assimilation^1^. This is effectively demonstrated by a version of White's Illusion (White, 1979), where the test patches are not as wide as they are tall (Figure 1 right).

Theories that aim to explain lightness illusions can be broadly categorized into low-level and higher-level accounts. Higher-level theories argue that scene interpretation is necessary to account for lightness illusions, where cortical processing of surface curvature, depth, and transparency are known to influence perceived reflectance (Knill and Kersten, 1991). For instance, Schirillo et al. (1990) demonstrated that lightness perception is dependent upon depth cues. Given that depth perception is thought to be a cortical function, higher-level areas must be recruited when perceiving reflectance. In 1999, Gilchrist et al. (1999) established the Anchoring Theory of lightness, where perceived reflectance of a patch is “anchored” to the highest luminance value within the retinal image (global information) and is also “anchored” to luminance values in surface groups that share commonalities such as being situated within the same depth plane (local information). Another notable high-level theory is Anderson (1997)'s Scission Theory, based upon the principle that a visual scene is split into different causal layers of reflectance, transparency, and illumination (the amount of light incident on a surface), to determine the surface properties of a homogenous area. While these high-level theories are able to offer consistent explanations for a variety of complex lightness phenomena, our aim in this paper is to quantify the performance of low-level models whose computations do not require higher-level scene interpretation. In the interests of providing a succinct quantitative account of a range of lightness phenomena, we apply Occam's Razor, emphasizing the capability of low-level theories to deliver improved modeling precision with greater parsimony.

Low-level theories concentrate on filtering operations and statistical image properties as the key explanation behind many lightness illusions. The main principle underlying low-level theories is that of image reconstruction: that lightness is inferred by reconstructing the most probable source image using filtering operations (Blakeslee and McCourt, 1999; Dakin and Bex, 2003). The filters concerned are considered to reside in early stages of the visual hierarchy such as the retina, LGN, and/or V1. Blakeslee and McCourt (1997) designed a low-level model using a multi-scale array of two-dimensional Difference of Gaussian filters (DOG). The isotropic filters in this model approximated retinal ganglion or LGN single cell function. The DOG model was able to account for the contrast effect shown in the SCI but not the assimilation observed in White's Effect. To account for assimilation, Blakeslee and McCourt (1999) extended this model to include anisotropic filters (oriented difference of Gaussians, or ODOG filters) that were pooled non-linearly. These orientation selective filters best approximate V1 functions, shifting the focus of the model from pre-cortical to cortical operations to account for a larger set of lightness illusions. Shortly after this, Dakin and Bex (2003) introduced an isotropic filter model that reweighted filter outputs using spatial frequency (SF) properties found in image statistics. Using a series of center-surround, Laplacian of Gaussian filters, they demonstrated that low SF structure is an essential ingredient of two well-known lightness illusions: White's Effect and the Craik-Cornsweet-O'Brien Effect (O'Brien, 1958; Craik, 1966; Cornsweet, 1970). Dakin and Bex (2003) demonstrated that orientation selective filters were not required to successfully model assimilation effects, and highlighted the importance of weighting or normalization schemes within these low-level models.

Since Dakin and Bex's paper, focus on statistical image properties (Corney and Lotto, 2007) and on post-filtering operations that weight the relative filter outputs (Robinson et al., 2007) has intensified in the context of low-level lightness models. Corney and Lotto (2007) demonstrated contrast and assimilation effects using an approach inspired by image statistics, training an artificial neural network with virtual scenes that possess naturalistic structure. In contrast to Dakin and Bex (2003) who made statistical relationships explicit through weighting operations, Corney and Lotto (2007) trained an artificial neural network to implicitly learn the relationships between images and their underlying statistics. In the same year, Robinson et al. (2007) focused on applying different normalization schemes to improve predictions using the ODOG model. Normalization is commonly used as a weighting scheme to smooth distributions and scale all values to a baseline magnitude (usually 1). Robinson et al. (2007) focused on applying two different normalization schemes to the ODOG model: local normalization of filter outputs (LODOG) and spatial frequency-specific local normalization (FLODOG). In LODOG and FLODOG, parameters of the normalization function (such as normalization window size) were adjusted to produce different model predictions. Robinson et al. (2007) systematically tested ODOG, LODOG, and FLODOG on a catalog of 28 stimuli, 27 of which are known to induce illusions of contrast or assimilation in human observers. While ODOG was able to predict only 13 illusions in the correct direction, the best performing LODOG model was able to predict 18. FLODOG proved the most effective, correctly predicting 24 lightness illusions with an optimal parameter set.

**Figure 2:**
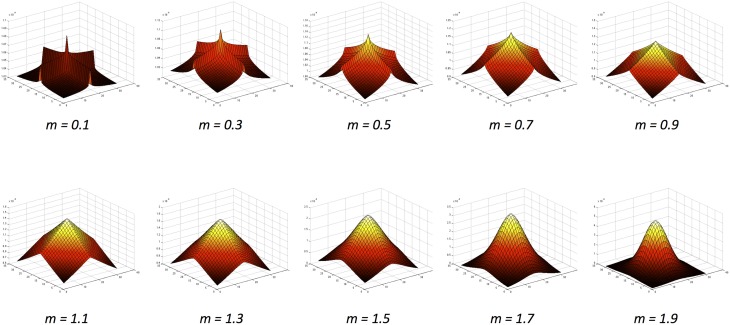
**The exponential function family (Basu and Su, 2001) with increasing values of the** ***m*** **exponent**.

Here we extend the literature using an approach inspired by natural image statistics. As established by Dakin and Bex (2003), the underlying distribution of structural properties present in natural images can greatly influence lightness judgments. Natural images share common underlying statistics, regardless of their origin (Zhu and Mumford, 1997a,b). For example, contrast histograms for natural images are skewed toward lower contrasts and have an exponential tail (Field, 1987; Ruderman and Bialek, 1994). Basu and Su (2001) investigated filters that encode the distribution of contrasts over different spatial frequencies. They concluded that exponential distributions provide a better fit in representing the underlying power distributions of natural images than the Gaussian kernels that have been used in the models described above. By employing exponential filters of different sizes and shapes within a computational model, we represent the profile of contrast statistics present in natural images and observe how these may influence the direction and magnitude of a set of lightness illusions. These filters have x- and y-axis symmetry, ranging from ridged, ‘peaky’ distributions to flatter, more rounded distributions (illustrated in Figure 2).

The exponential filters we explore in this study are offered as another kind of inhibitory mechanism, since the image filtered by the exponential function is subtracted from the original image. As such, this model shares much in common with other filtering approaches, such as ODOG (Blakeslee and McCourt, 1999). Indeed, this filtering approach bears similarity to the extra classical surround model of Ghosh et al. (2006) and is most similar to the filtering approach of Shapiro and Lu (2011), with the exception of the shape of the surround.

While the filters in ODOG (and variants) approximate the functioning of orientation-selective V1 cells, and while Difference or Laplacian of Gaussian filters approximate the operations of isotropic LGN or retinal ganglion cells, exponential filters, not unlike those forming the basis of our model, have been identified in H1 horizontal retinal cells (Packer and Dacey, 2002, 2005). Our model is predominantly motivated by the computer vision literature, where exponential filters have been shown to be excellent edge detectors as well as resilient to noise (Zhu and Mumford, 1997a). The level of biological plausibility in our model is not strongly emphasized, but we do identify possible neurobiological equivalents to the filters that we apply. Geisler (2008) illustrates responses to natural images of a sensor that has a receptive field profile similar to V1, where an exponential function shows a better fit over a Gaussian distribution. While there are parallels here in demonstrating that an exponential fit is better than Gaussian in terms of filter responses, the filters that we apply are not oriented V1-style filters. Therefore, we would not suggest any relationship between our model results and the involvement of cortical neurons.

Our study differs from that of Corney and Lotto (2007) in that we make statistical relationships explicit through filtering and normalization operations, instead of training an artificial neural network to implicitly learn the relationships between images and their underlying statistics. Our method is similar to that of Dakin and Bex (2003), in that we both capitalize on the properties of image statistics to reconstruct the final image. In our method, we employ exponential shape filters that are based on image statistics. In Dakin and Bex (2003), the authors split an image into different spatial frequencies (SFs) using band-pass filters. The distribution of SFs was then reweighted to match that which occurs in natural scenes. In our model and in that of Dakin and Bex (2003), the filters are designed to extract the most salient features while being robust to noise (Basu and Su, 2001). In this way, both of our studies align with the predictive coding principle by Srinivasan et al. (1982)—that by exploiting the spatial correlations of natural scenes, early visual systems are much better able to handle noise in the environment.

In the current study, we set out to investigate how well an exponential model is able to predict human data in response to a large battery of 28 lightness illusions previously used to test ODOG and its derivatives (Blakeslee and McCourt, 1999, 2001, 2004; Blakeslee et al., 2005; Robinson et al., 2007). We apply exponential filters with a range of different shapes and sizes to an input image, with and without normalization of varying spatial extent. The outputs of this model are taken as predictions of lightness both for single filters and for multiple-filter combinations.

## 2. Materials and methods

### 2.1. Stimuli

**Figure 3:**
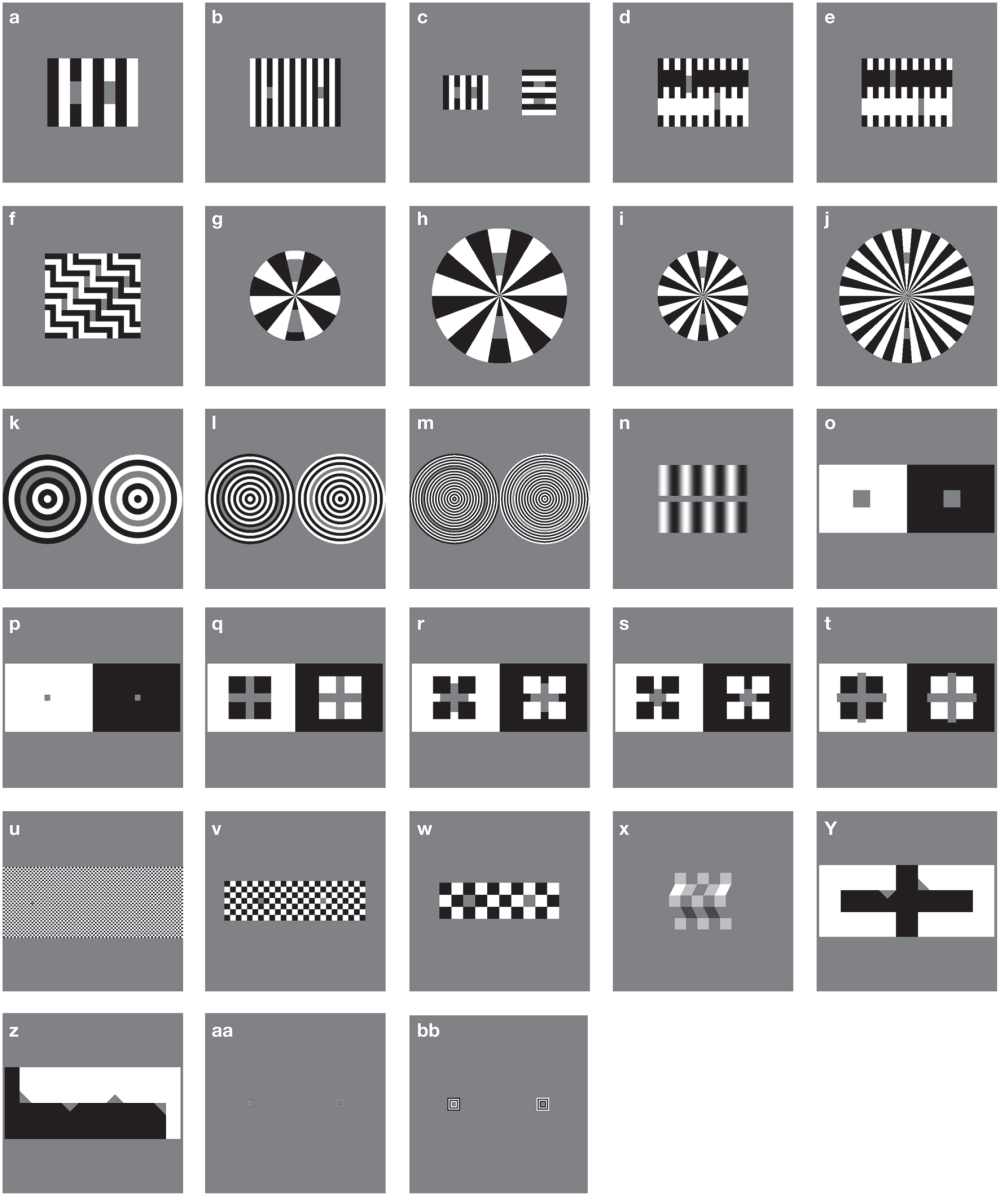
**Illusions tested, replicated from Robinson et al. (2007)**. Each letter refers to an individual stimulus.

A standard battery of 28 figures known to produce particular lightness effects was used as a stimulus set in this study (see Robinson et al., 2007). Each stimulus (with the exception of the Benary Cross) involves a pair of uniform, mean luminance target patches, each surrounded by details with the opposite contrast polarity. Stimuli are illustrated in Figure 3, reproduced from Robinson et al. (2007). All stimuli are 512 × 512 pixels in size. Each stimulus is listed below in Table 1 with original sources and comparative results reported for human responses where available. Table 1 also includes the reported illusion direction by humans as the patch perceived as the lightest within the image and the corresponding classification of the predominant effect as contrast or assimilation.

**Table 1:** **Stimuli with original sources, reproduced results (for strength comparison) and illusion direction reported by humans**.

**Figure**	**Original source**	**Reproduced results**	**Human Direction**	**Contrast (C) or Assimilation (A)?**
a	White, 1979	Blakeslee and McCourt, 1999	Left	A
b	White, 1979	Blakeslee and McCourt, 1999	Left	A
c	Robinson et al., 2007		Top	A
d	Anderson, 2001	Blakeslee et al., 2005	Right	A
e	Howe, 2001	Blakeslee et al., 2005	No illusion	N/A
f	Clifford and Spehar, 2003		Left	A
g	Anstis, 2003		Bottom	A
h	Anstis, 2003		Bottom	A
i	Anstis, 2003		Bottom	A
j	Anstis, 2003		Bottom	A
k	Howe, 2005		Right	A
l	Howe, 2005		Right	A
m	Howe, 2005		Right	A
n	McCourt, 1982	Blakeslee and McCourt, 1999	Area between black	C
o	Chevreul, 1839	Blakeslee and McCourt, 1999	Right	C
p	Chevreul, 1839	Blakeslee and McCourt, 1999	Right	C
q	Pessoa et al., 1998	Blakeslee and McCourt, 1999	Left (Right in original)	C
r	Todorovic, 1997	Blakeslee and McCourt, 1999	Right	A
s	Todorovic, 1997	Blakeslee and McCourt, 1999	Right	N/A
t	Pessoa et al., 1998	Blakeslee and McCourt, 1999	Right	A
u	De Valois and De Valois, 1988	Blakeslee and McCourt, 2004	Right	A
v	De Valois and De Valois, 1988	Blakeslee and McCourt, 2004	Right	A
w	De Valois and De Valois, 1988	Blakeslee and McCourt, 2004	Left	C
x	Adelson, 1993	Blakeslee and McCourt, 2001	Bottom	C
y	Benary, 1924	Blakeslee and McCourt, 2001	Left	N/A
z_2−1_	Todorovic, 1997	Blakeslee and McCourt, 2001	Second in 1–2	N/A
z_4−3_	Todorovic, 1997	Blakeslee and McCourt, 2001	Fourth in 3–4	N/A
aa	Bindman and Chubb, 2004		Left	A
bb	Bindman and Chubb, 2004		Left	A

The majority of images exhibit assimilation effects, with contrast effects demonstrated by figures *n*, *o*, *p*, *q*, *w*, and *x*. In some cases, target patches have equal bordering white and black areas, making it difficult to establish whether a lightness effect should be defined as a contrast or assimilation effect (as in stimulus *s*). Stimuli *y* and *z* demonstrate opposing illusion directions for patches with identical bordering surrounds, presenting both contrast and assimilation effects simultaneously. In most cases, illusory effect directions reported in the original articles have been replicated in follow-up studies by Blakeslee and McCourt (used here and in Robinson et al., 2007 for direct strength comparisons). However, due to slight differences in methodology, stimuli *e* and *q* demonstrate discrepancies between the two sets of human data. In these cases, we follow the convention of Robinson et al. (2007) to allow for easy comparison between their models and those described here.

As each stimulus involves 2 (or more) uniform, mean luminance target patches, each surrounded by details with the opposite contrast polarity, the lightness effects observed on these patches are expected to be equal and opposite. Our model's predictions regarding the presence of contrast or assimilation effects are made by taking mean lightness values from the largest rectangular patch inside the bounds of the target areas (matched for size) and subtracting the values for the patch that appears darker from those for the lighter. For stimulus *n*, (“grating induction”), we select rectangular areas that are 26 pixels wide to the left and right of center for our prediction comparison (0.4 of the spatial period of the grating), while maintaining the same patch height as Robinson et al. (2007).

### 2.2. Model

Our model consists of two-stages: (1) linear filtering using exponential functions (2) non-linear divisive normalization by coefficient of variation. Although the details of each stage may vary, this linear-nonlinear modeling method is commonly used to model physiology (Schwartz and Simoncelli, 2001; Nykamp and Ringach, 2002). Once the two stages of the model have produced lightness values at each pixel location of each target patch, we produce a prediction by calculating the mean difference over the target patches and applying linear scaling. Details of each step in the model and on calculating the comparison metric are described below.

#### 2.2.1. Filtering

The set of exponential filters we apply are taken from Basu and Su (2001). These exponential filters are two dimensional in shape and possess x-symmetry, y-symmetry, and symmetry with respect to the origin. They have unit volume and take the form:

(1) $$g(x)=\frac{1}{K_{1}}exp^{-K_{2}|x{|}^{m}}$$

where K_1_, K_2_, and m are all positive constants. The m exponent corresponds to the shape of the filter. The normalization or scaling factor K_1_ is calculated using K_2_ and m as follows:

(2) $$K_{1}=(1/K_{2}^{1/m})(1/m)\Gamma (1/m)$$

where constant K_2_ is a function of the variance of *g*(*x*), which denotes the size of the filter. Γ(*x*) is the Gamma function defined as:

(3) $$\Gamma (x)=\int_{0}^{\infty }t^{\left(x-1\right)}exp(-t)dt$$

Figure 2 illustrates the variety of exponential filter shapes. When m is small, the exponential filter is described as having “high kurtosis,” showing a sharper peak with more prominent ridges. When m is large, the exponential filter has “low kurtosis,” being flatter and rounder with smoother ridges. A special case is formed when m = $\frac{1}{2\sigma^{2}}$, where the function becomes a Gaussian with added rotational symmetry.

Each filter of a specific size and shape is applied to every pixel within the image. The size of the filter affects the information that is gleaned from an image. Smaller filters (high spatial frequencies or SFs) show better responsiveness but are less resilient to noise. Larger filters (low SFs) blur a lot of information, essentially losing information present in the images, but cope better with noise. There is a trade-off between selecting precise information and having greater resilience to noise, which is where scale selection comes in. The most appropriate filter selection finds the right compromise between these two factors, taking the smallest scale with the most reliable response.

A small amount of Gaussian noise is added to the image (0.1%) before filtering. Adding noise to the image is to avoid divide-by-zero errors when implementing divisive normalization. We are aware of other approaches to avoid divide-by-zero errors, such as adding a constant to the denominator term (Cope et al., 2013).

**Figure 4:**
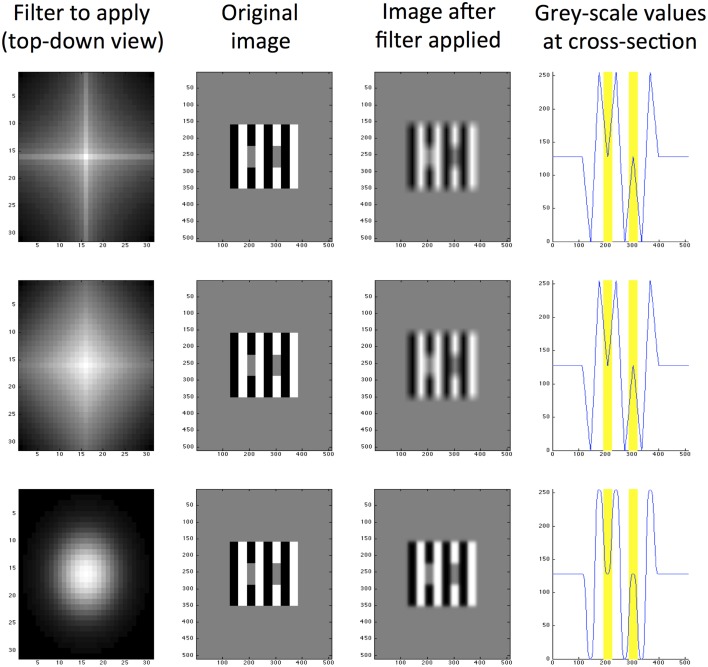
**Exponential filters applied to White's illusion, all with size** ***K*****_2_ = 5**. The top row shows a filter with high kurtosis (*m* = 0.5), the middle row shows a medium kurtosis filter (*m* = 1.0) and the bottom row shows a low kurtosis filter (*m* = 2.0). From left to right, column 1 is a top-down view of the filter shape, column 2 is the original image (of size 512 × 512 pixels), column 3 is the same image filtered and column 4 is a cross section of grayscale values through row *y* = 250 pixels (where 0 represents black and 255 represents white). The locations of target patches are highlighted yellow in the final column.

Responses are then convolved to create a filtered image of the same dimensions as the original input. The filtered convolved image is subtracted from the original image as the final step in processing. We explore a range of different filter shapes and sizes and produce a set of filtered images for every size and shape of filter. We use 10 filter sizes ranging from 5 pixels to 95 in increments of 10. The filter shapes range from 0.1 to 1.9 in increments of 0.2. Figure 4 illustrates the result of applying three example filters with different shape parameters to White's Illusion. The predictive success of this particular filter size is well-demonstrated for this particular image, regardless of filter shape. The bottom row in Figure 4 demonstrates a close approximation to the Gaussian filter, which in this case is able to predict the direction and magnitude of White's Effect. This filter differs from the DOG filters used by Blakeslee and McCourt (1997)'s model in two key ways. Firstly, Blakeslee and McCourt use a Difference-of-Gaussian (DOG) filter, rather than an approximate Gaussian pictured here. Secondly, Figure 4 demonstrates a single filter operation, rather than a bank of filters used by Blakeslee and McCourt (1997).

#### 2.2.2. Normalization (optional)

After applying a specific contrast filter with shape *m* and size K_2_ to each pixel location in the image, we optionally normalize the filter outputs. Normalization is not only useful in its primary function of constraining the dynamic response range of image filters, but is also beneficial for generating a faithful representation of image contrast. Following (Bonin et al., 2005), at each image location we divisively normalize the linear filter output by the output of a suppressive field, which computes the statistics of filter outputs surrounding the image location of interest. Bonin et al. (2005)'s normalization method, referred to as contrast gain control, is closely related to that found in the LGN and so we apply it here as a biologically plausible method for normalization in pre-cortical areas. In contrast to Bonin et al. (2005), who take the local root-mean-square contrast as the suppressive field, we divide filter responses by the local coefficient of variation. The local coefficient of variation is inversely related to local Weibull statistics and as such is diagnostic of local image structure. Divisive normalization by the local coefficient of variation amplifies local image contrast. Similarly to Bonin et al. (2005), we compute normalized filter outputs using the following formula:

(4) $$V=V_{max}\frac{g(x)}{c_{50}+c_{local}}$$

where *c*_50_ determines the strength of the suppressive field, *V*_*max*_ is the maximum response of the filter to the image, and *g*(*x*) is the filtered response defined above. Finally, *c*_*local*_ is the local coefficient of variation:

(5) $$c_{local}=\frac{\sigma }{\mu }$$

*c*_*local*_ is calculated based on the mean (μ) and the size of the suppressive field (σ) that is used as one of the parameters in our normalization step. The σ parameter specifies the size of the suppressive field compared to the size of the receptive field. When σ = 1, the size of the suppressive field is equal to that of the receptive field. When σ = 2, the size of the suppressive field is twice that of the receptive field.

#### 2.2.3. Analysis metrics

For each stimulus that we analyze, we take the resultant values (denoted as *R*) from the filter-only output (step 1) or from normalized output (step 2) with either σ = 1 or σ = 2 (as described above, σ represents the size of the suppressive field, as a proportion of the receptive field). We refer to σ = 1 as short-range normalization, where the suppressive field is the same area as the receptive field. σ = 2 is referred to as long-range normalization, where the suppressive field is twice the size of the receptive field. Within each image, we compare values over the two areas that have been assigned to be target patches (see Section 2.1). The lighter patch (as established in human experiments) is assigned to be patch *A* and the darker patch is assigned to be patch *B*. Mean values are obtained for both target patches before the mean of patch *B* is subtracted from the mean of patch *A*. Because patch *A* is assigned to be the lighter patch, a prediction in the correct direction is indicated by a positive value, whereas an incorrect prediction is negative. A value of zero indicates no difference in patch lightness values and therefore no illusion.

To compare resultant values, we scale the difference between target patches to the strength of White's Illusion for ease of comparison. The magnitude of White's illusion is denoted as *R*_*a*_. This means that all resultant values are scaled to the strength (or magnitude) of White's illusion. A resultant value of 1 is then interpreted as having identical illusory strength to White's illusion. A value greater than 1 indicates the illusion is stronger than White's, and a value less than 1 (and above 0) indicates the illusion is weaker than White's. Although any stimulus could have been selected for comparative purposes, we follow Robinson et al. (2007)'s convention by selecting stimulus *a* as our comparative figure.

(6) $$R=(\bar{A}-\bar{B}).\;/|R_{a}|$$

We also calculate the difference between model predictions and human results (where available) to quantify how well-different model configurations match human data. We do this by subtracting the human result *R_human_* from the model result *R_model_* for stimuli from *a* to *bb* for which human results are available, and calculating the root mean square error (*RMS_error_*). The smaller the *RMS_error_* value, the better the model matches human data, and the greater the predictive accuracy of the model in terms of illusion magnitude or strength.

(7) $$RMS_{error}=\sqrt{\frac{1}{n}\sum_{a}^{bb}(R_{model}-R_{human}{)}^{2}}$$

When combining the outputs of two filters α and β of different sizes or shapes, we simply sum the difference in mean responses to the light and dark patches separately for each filter (removing scaling to figure *a*):

(8) $$R^{\alpha \beta }=R^{\alpha }+R^{\beta }$$

## 3. Results

**Figure 5:**
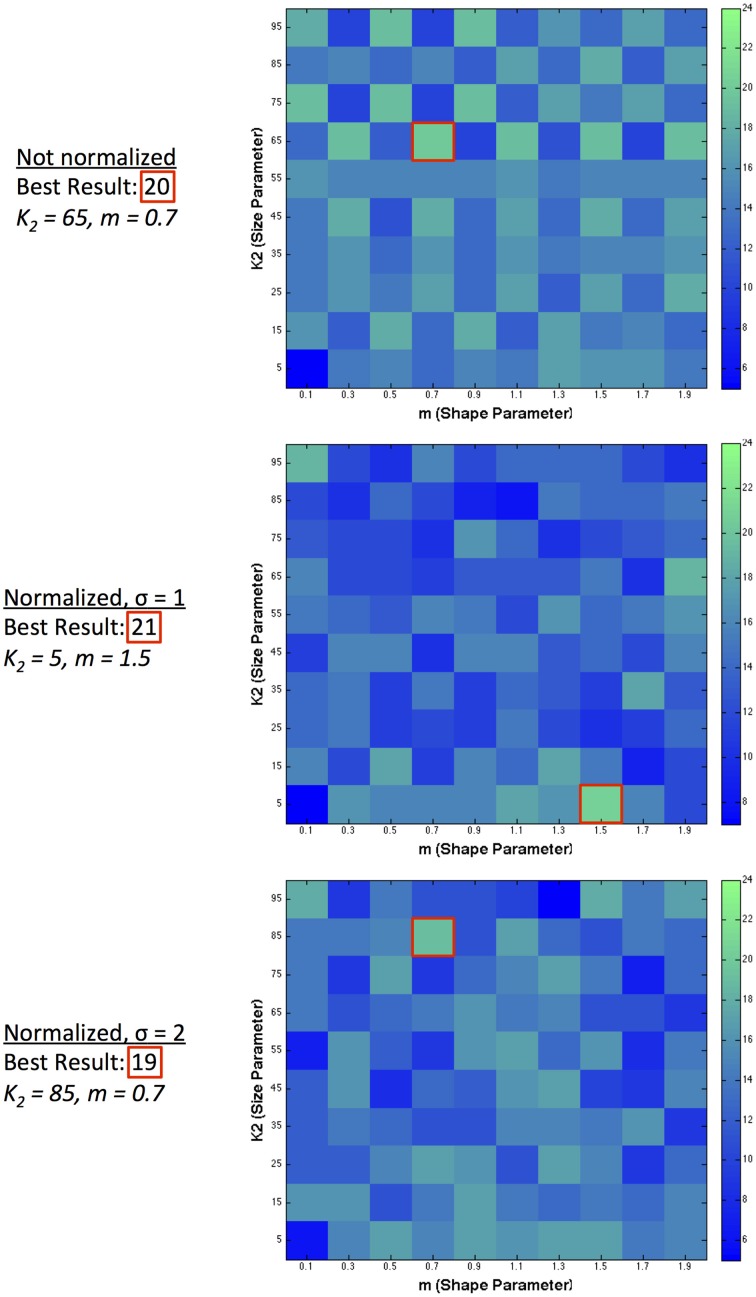
**Single filter predictions over 10 different shapes and 10 different sizes**. The number of correct illusion directions predicted for different model configurations using a single filter.

We assess the performance of our model in two ways: the number of predictions in the correct direction, and also how closely the predicted values match the scaled human data on illusion magnitude. We exclude figure e from our analysis, given that no illusion direction is reported for humans. Figure 5 illustrates the number of illusion directions correctly predicted (out of a maximum possible of 27) using a single filter over a range of 10 filter shapes and 10 filter sizes. For figure z, there are two predictions, annotated as *z*_2−1_ and *z*_4−3_, for comparing the two left patches and the two right patches in the image, respectively. We take a correct result to be when (*z*_2−1_ + *z*_4−3_)/2 > 0. *RMS_error_* is also calculated using the average over these two comparisons. We show predicted results for various model configurations: with no normalization, and with 2 ranges of local normalization (σ = 1 and σ = 2). With no normalization, the highest number of correct direction predictions made by a single filter was 20 illusions using a large-sized filter with medium kurtosis. With short-range normalization (σ = 1), the highest number of correct direction predictions made by a single filter was 21 illusions (present in a small-sized filter with high kurtosis). With an increased normalization range (σ = 2), the best prediction result was slightly lower at 19 out of 28.

Table 2 lists the results for the best performing size and shape filter in terms of the difference of the mean values over the target patches. As mentioned above, we exclude figure e from our results because no illusion is reported in human results. We report values for *z*_2−1_ and *z*_4−3_ (in gray) and take the average of these two as our prediction for z, maintaining a single value prediction per illusion. In Table 2 we also reproduce results from Robinson et al. (2007) for the ODOG, best LODOG, and best FLODOG model alongside human scaled results for direct comparison. Predictions in the correct direction are shown in bold and tallies of the number of these correct predictions are presented at the bottom. For each model, we also list the *RMS_error_* that represents how well the model's predictions match the magnitude of human results.

**Table 2:** **Model results for the best single filter with and without normalization alongside ODOG and unscaled human results**.

**Figure**	**Shorthand Name**	**Human Scaled**	**ODOG**	**LODOG *n* = 2**	**FLODOG *n* = 2s *m* = 0.5**	**Exp model Single filter No norm**	**Exp model Single filter Norm σ = 1**	**Exp model Single filter Norm σ = 2**
a	WE-thick	1	**1.00**	**1.00**	**1.00**	**1.00**	**1.00**	−1.00
b	WE-thin-wide	1.1	**2.08**	**2.08**	**2.52**	**19.36**	**0.73**	**1.18**
c	WE-dual		−0.30	**1.36**	**1.93**	−8.57	**0.28**	−0.49
d	WE-Anderson	1.54	−0.15	−0.30	−0.43	−1.68	−0.37	**0.95**
f	WE-zigzag		−0.51	−0.76	**1.26**	**55.52**	**0.42**	−1.69
g	WE-radial-thick-small		−0.67	−0.39	**0.46**	**0.52**	**0.18**	**0.16**
h	WE-radial-thick		−0.41	**0.01**	**0.18**	**0.16**	−0.16	**1.09**
i	WE-radial-thin-small		−0.34	**0.21**	**2.74**	**2.32**	**0.28**	−1.00
j	WE-radial-thin		−0.22	**0.83**	**3.24**	**0.52**	**0.58**	**1.91**
k	WE-circular1		−0.82	−1.04	**0.28**	**1.24**	**0.22**	**0.52**
l	WE-circular0.5		−0.53	−0.67	**1.84**	−2.84	**0.67**	**2.40**
m	WE-circular0.25		−0.38	−0.49	**3.64**	−2.15	**0.55**	−1.30
n	Grating induction	1.49	**2.03**	**1.69**	**0.66**	**0.20**	**0.18**	−0.30
o	SBC-large	2.72	**4.75**	**7.56**	**3.96**	**4.01**	**3.09**	**0.75**
p	SBC-small	4.73	**6.22**	**14.94**	**5.96**	**8.79**	**4.52**	**7.02**
q	Todorovic-equal	0.53	−0.36	−0.26	**0.08**	**0.19**	**0.02**	**1.33**
r	Todorovic-in-large	0.57	**0.49**	**0.55**	**0.39**	**0.03**	**0.20**	−1.00
s	Todorovic-in-small	1.05	**0.80**	**0.95**	**1.08**	**0.32**	**0.19**	**0.80**
t	Todorovic-out	0.37	**0.35**	**0.38**	**0.03**	**0.34**	−0.07	**1.86**
u	Checkerboard-0.16	1.78	**1.10**	**0.94**	**8.03**	−0.34	**0.33**	**2.69**
v	Checkerboard-0.94	0.68	**0.40**	**0.35**	−4.89	**20.26**	−0.19	**3.93**
w	Checkerboard-2.1	1.36	**0.69**	**0.60**	−1.48	**0.61**	**0.05**	**0.77**
x	Corrugated Mondrian	2.6	**0.95**	**0.91**	**0.12**	**12.92**	−0.02	**2.32**
y	Benary cross	2.2	**0.09**	**0.06**	**0.05**	−559.23	**0.23**	−1.94
z_2−1_	Todorovic benary 1–2	2.86	−0.12	**0.55**	**0.11**	−1408.10	**0.23**	**1.77**
z_4−3_	Todorovic benary 3–4	2.28	−0.12	**0.58**	**0.14**	**1383.70**	**0.14**	**7.16**
z avg	Todorovic benary average	2.57	−0.12	**0.57**	**0.13**	−12.20	**0.18**	**4.47**
aa	Bullseye-thin		−0.74	−0.35	**0.54**	**0.18**	**0.02**	**3.31**
bb	Bullseye-thick		−0.77	−0.38	**0.07**	**1.16**	−1.49	**1.59**
**Total correct**			13	18	24	20	**21**	19
***RMS_error_***			1.29	1.80	2.56	140.59	**1.32**	1.85

*The result reported for z is the average of z1 and z2 listed in gray. Bold values indicate predictions in the correct direction. Tallies of correct predictions are presented at the bottom for each model. The most successful single filter result using the exponential filter model has its tally highlighted in bold*.

Table 2 shows that performance was maintained (in terms of number of correct direction predictions) when going from raw filter output to short-range normalized results for single filter predictions. Normalized results provided predictions with much smaller magnitudes of lightness illusions, as we would expect. Across predictions of both direction and magnitude, normalized results with σ = 1 provided the best predictions for single filters, showcasing the highest number of correct direction predictions (21) and reasonable magnitudes for these predictions (indicated by a substantially reduced *RMS_error_* compared to filter-only output). Indeed, in this case *RMS_error_* shows an accuracy of prediction that is matched only by the small values of the ODOG model, which fares considerably less well in terms of number of correct direction predictions (13). The *RMS_error_* increased when the normalization range was extended to σ = 2, where only 19 correct direction predictions were made.

**Figure 6:**
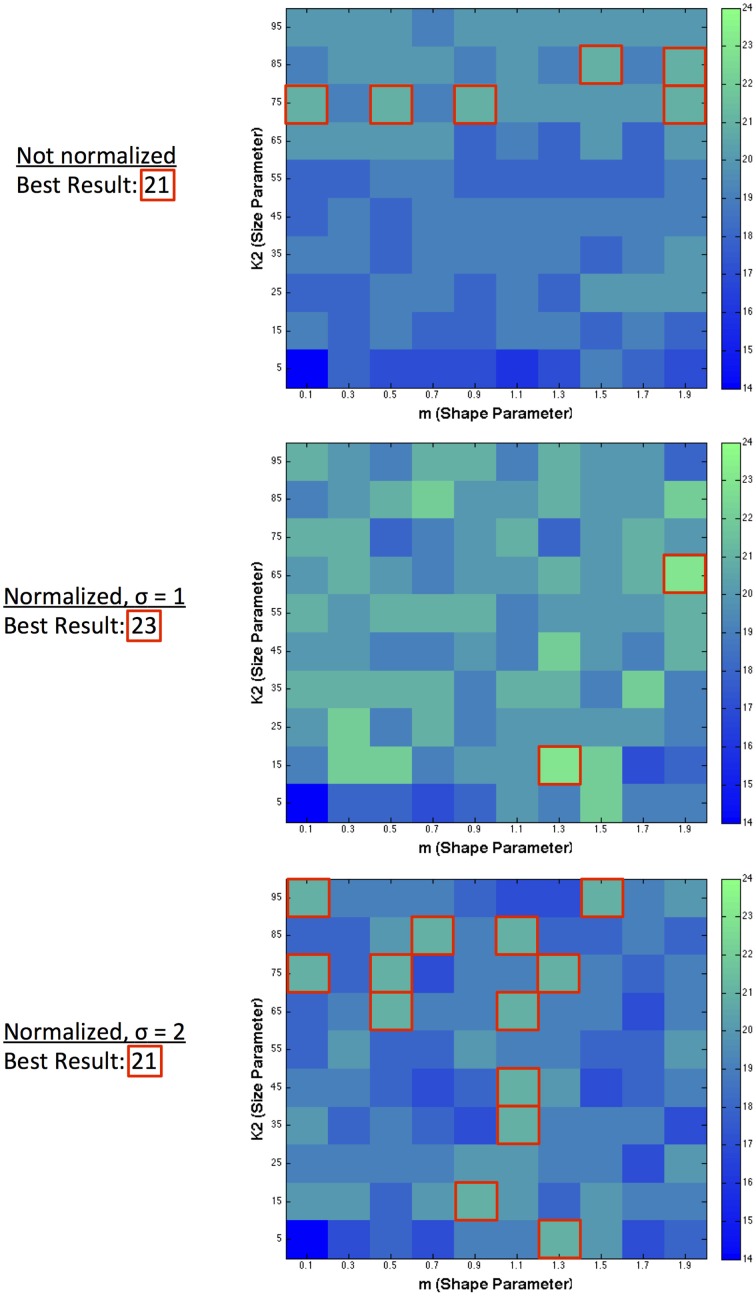
**Dual filter predictions**. Highest predictive success when combining a filter of specified size and shape with any other size and shape filter.

The results presented so far have demonstrated the capability of single filter predictions. We also combined multiple filters to observe the possibility of improving predictive success. Figure 6 shows the result of combining pairs of filters together, taking a particular size and shape filter and combining it with the best possible match to maximize the number of correct directions predicted. The best result across all environments (normalized and filter-only), for dual filter combinations was 23 correct directions. The best resultant combinations in terms of maximizing the number of correct prediction directions occurred for a number of filter pairings within different environments. In the filter-only environment, the best filter pair combinations occurred across 6 different large sized filters ranging from high to low kurtosis. For normalized filters with σ = 1, the best filter pair was with a small sized filter with medium kurtosis and a medium sized filter with low kurtosis. For normalized filters with a larger range of normalization (σ = 2), the best pairings occurred across a range of filters with medium kurtosis over various sizes, or were large in size and had low to medium kurtosis.

**Figure 7:**
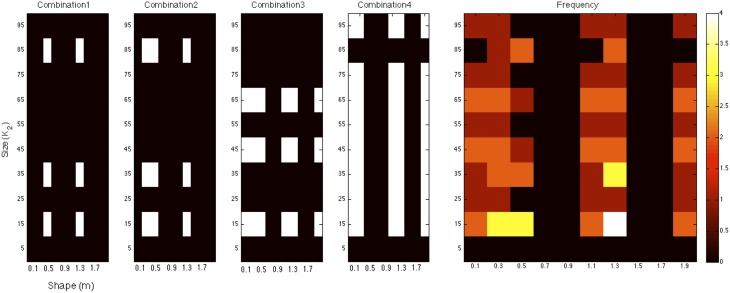
**The four filter combinations that achieve the maximum of 24 correct illusion direction predictions for the exponential filter model**. These combinations were found for short-range, normalized filters. The filters across all four combinations were tallied and the frequency of these is presented on the right.

We extended our multi-filter analysis to allow for the combination of any number of size and shape filters to determine whether an optimal combination of multiple filters exists. Using an ordered search sequence over the space of all possible shape and size filter combinations, we found that the maximum predictive success (in terms of illusion direction) that the model was able to achieve was 24 out of 27. This value represents the upper bound of performance of this exponential filter model and was found for the set of short-range normalized filters. Figure 7 illustrates the four filter combinations that achieve the maximum of 24 correct illusion direction predictions for the exponential filter model. This was found for the set of normalized (σ = 1) filters. The filters across all four combinations were tallied and the frequency of these is presented on the right. A minimum of 6 filters was required to reach the best prediction as shown in combination 1. These were filters of size *K*_2_ = (15, 35, 85) and shape *m* = (0.5, 1.3). Combinations 2–4 in Figure 7 show the other filter combinations for which 24 illusion directions were correctly predicted. We see that a spread of different size and shape filter combinations is required to produce the best predictive performance. Certain filters are found to be informative whereas others are found to be consistently uninformative. Looking at the frequency of specific size and shape filters across all five most successful combinations, we see that filter (*K*_2_ = 15, *m* = 1.3) is common across all filter arrangements. It is also evident that the organization of multiple filters is distributed across the parameter space.

## 4. Discussion

In this study, we applied a series of exponential filters differing in scale and shape to a set of lightness illusions that have previously been tested with Oriented Difference-of-Gaussian (ODOG) filters and associated models. The exponential model far outperforms the early ODOG models, and demonstrates predictive capabilities that match the successes of more recent elaborations of these models—LODOG and FLODOG—that incorporate local normalization post-filtering. Using a single filter, the direction of 21 (out of a possible 27) illusions can be predicted successfully. Using a two-filter combination, the predictive success of the model increases to 23. Extending the model to include any number of combined shape or size filters allows us to define the maximum capability of this model as 24 correct illusion direction predictions. Our results show that a low-level filtering model based on exponential filters can account for a large number of lightness illusions without requiring orientation-selective filters.

Comparing our work to the current literature, we highlight that existing models are restricted to filters of a specific shape (either DOG or LoG). We wanted to explore the effect of variation in the shape of the filters, which remains fixed in existing models. Our aim was not to emphasize stronger prediction performance, but to investigate whether filters inspired by image statistics can provide predictions on par with current state-of-the-art models. We have shown that this is indeed the case, where Gaussian-shaped filters do not provide the best predictability for the illusion set under all circumstances.

While the 28 stimuli used in this study feature substantial differences, one pertinent respect in which they vary is the induction of contrast or assimilation. Six of our illusions can be classified as predominantly contrast effects, whereas 18 primarily produce assimilation, with 4 illusions unclassifiable (see Section 2.1). Our best single-filter model was able to achieve 5/6 and 13/18 accuracy for contrast and assimilation effects, respectively, showing its ability to deal effectively with both classes of effect.

Among our catalog of illusions there are several sets of images that vary principally in terms of SF. These not only include low and high SF versions of White's Effect (*a* and *b*) and the SCI (*o* and *p*) as highlighted in Figure 1. Variations in SF are also seen for radial White's Effect (figures *g* through to *j*), circular configurations of White's Illusion (figures *k*, *l*, and *m*), the Checkerboard illusion (*u*, *v*, and *w*) and Bullseye figures (*aa* and *bb*). In Table 2 (column 3), we list values of illusion magnitudes where human data is directly comparable with various SF configurations of the same illusion (reproduced from Robinson et al. 2007). Such comparisons are available for White's illusion (*a* and *b*), the SCI (*o* and *p*) and the Checkerboard illusion (*u*, *v*, and *w*). We draw direct conclusions for the performance of our best single-filter model to these figures. For the remaining figures with no directly comparable human data, we make observations based on the general rule that higher spatial frequencies yield greater effects. Our best single-filter model (normalized with σ = 1) predicts the correct direction of illusion for both high and low SF versions of White's illusions (stimuli *a* and *b*) and of the SCI (figures *o* and *p*). In the case of the SCI the model can also account for the change in the size of the illusion as a function of SF, successfully predicting a larger effect at higher SF. However, in conflict with the human data, a reduction of the effect at higher SF is predicted for White's illusion. The Checkerboard illusion is an interesting case where the direction of the effect flips from assimilation to contrast for human observers when the visual angle of checkerboard squares is greater than approximately 1° of visual angle. Our best single-filter model is able to successfully account for two out of three illusion directions, with an appropriate increase in magnitude when comparing the lowest (*w*) and highest (*u*) SF versions. Despite an incorrect direction being predicted for figure *v*, the model correctly predicts a reduction in magnitude compared with *u*. Comparing the performance of our model to the best ODOG variants, we see that only ODOG and LODOG are able to account for all variations of correct illusory magnitudes where human data is available, performing with 5/5 correct relative magnitudes (for comparisons *b* > *a*, *p* > *o*, *u* > *v*, *w* > *v*, and *u* > *w*). The best performing model in terms of illusion direction, FLODOG, is able to successfully account for 3 out of a possible 5 illusory magnitudes consistent with SF. We conclude that our model is able to surpass that of FLODOG, with 4/5 illusion magnitudes that are commensurate with human data for both high and low spatial frequencies.

Reflecting on the best performance of the exponential model using a single filter, we note that two particular illusions that were predicted incorrectly—*t* (Pessoa et al., 1998); and *x* (Adelson, 1993)—warrant closer inspection. Stimulus *t* can be said to belong to the family of modified SCI figures from *q* to *t*. Figure *s* is a modified version of figure *o* (conventional SCI), where squares with opposite contrast polarity to the background are overlaid onto the target patch, creating equal boundaries of light and dark. Figures *r*, *q*, and *t* are modified versions of *s* with increasing crossbar lengths. The spectrum of figure arrangements from *q* to *t* demonstrate changes to figure-ground relationships in terms of object assignment, depth placement and scene segmentation. In figures *q*, *r*, and *s*, the target patch appears to be contiguous with the surrounding white or black regions (as in the SCI: see stimuli *o* and *p*), and is positioned behind black or white square occluders. However, in stimulus *t*, the figure that posed a problem for our most successful single filter model, a quite different depth arrangement is evident, as the target patch now forms a cross that appears to be the most proximal object, and no longer shares the same depth plane as the surround. The exponential model we adopt does not include higher-level information such as depth cues of occlusion. Depth information is also evident in the corrugated Mondrian (figure *x*), providing shadow cues that could be processed by higher cortical levels for lightness judgments. These results may be taken to support suggestions that some illusions may escape successful prediction by low-level mechanisms if their lightness depends on depth relationships (Schirillo et al., 1990).

While the ODOG model and its variants closely approximate the orientation selective operations in V1, exponential filters based on image statistics represent an efficient coding scheme that could be present in pre-cortical areas as early as the retina. The prevailing view in early work with lightness illusions was that they arose from retinal interactions, rather than cortical processing (Cornsweet, 1970; Todorovic, 1997). However, more recent research highlights the influence of higher-level mechanisms on our lightness perception (Adelson, 2000; Gilchrist, 2006). Using our model, we do not prescribe that filtering mechanisms alone can explain all lightness illusions. Instead, we set out to quantify the gap between what filtering operations can and cannot demonstrate. We propose that our exponential filtering model represents the first stage in a process of operations to estimate lightness. Later operations, such as those responsible for the scission of a scene into its component causal layers (Anderson, 1997) would occur post-filtering and normalization. The anchoring of lightness values to local and global context (Gilchrist, 2006) could occur within normalization operations or post-normalization. In our model's normalization step, the filtered image is first scaled to local responses (using local coefficient of variance) and then to the global maximum response within the image. This provides one of many approximations for the anchoring of lightness values.

**Figure 8:**
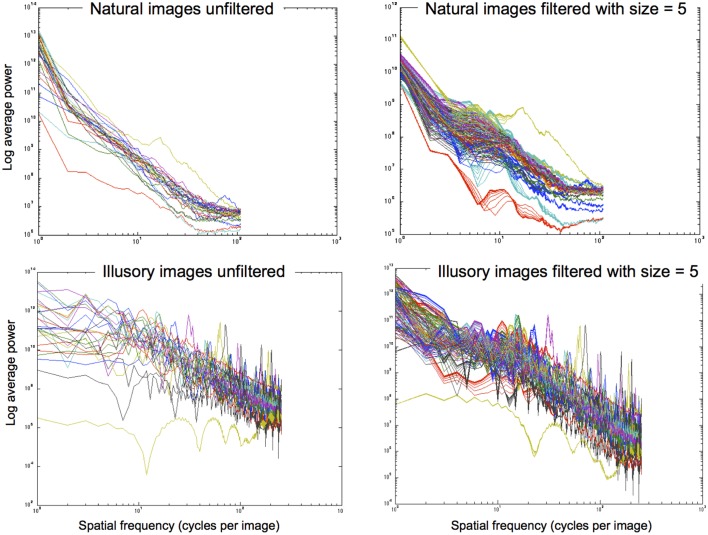
**Power spectra for images that are unfiltered (left column) and filtered with size = 5 pixels (right column). Top row:** 28 natural images. **Bottom row:** 28 illusory images.

The filtering approach we use reshapes contrast distributions toward those that best describe natural images using the exponential filter family. Similarly to Dakin and Bex (2003), we essentially reconstruct an image that represents the most probable naturally occurring source. By redistributing lightness values to more closely reflect the underlying statistical relationships of images within our environment, we can form predictions of perceptual lightness estimates that align with a large array of lightness illusions. Figure 8 illustrates the power spectra for a set of images that are unfiltered (left column) and filtered (right column) using different shape filters that are all of size 5 pixels. The top row illustrates power spectra for 28 natural images. From these graphs we can see that the power spectra for filtered natural images is quite similar to the power spectra for unfiltered natural images. The bottom row shows the power spectra for illusory images. The unfiltered images in the bottom left graph show a flatter power spectrum in the lower SFs than the filtered images in the bottom right graph. By applying these exponential filters, we see that they not only push the power spectra of illusory images toward that of natural images, reflecting the properties of image statistics. Applying these filters also boosts low SF information, hypothesized to be a driving factor in the perception of lightness illusions (Dakin and Bex, 2003). Dakin and Bex (2003) find that low spatial frequencies are primarily responsible for the Craik, Cornsweet, and O'Brien (CCOB) illusion that they study. The LoG filters that they apply boost this information when it is not present. From their results, Dakin and Bex (2003) hypothesize that low SF information may drive many illusions.

**Figure 9:**
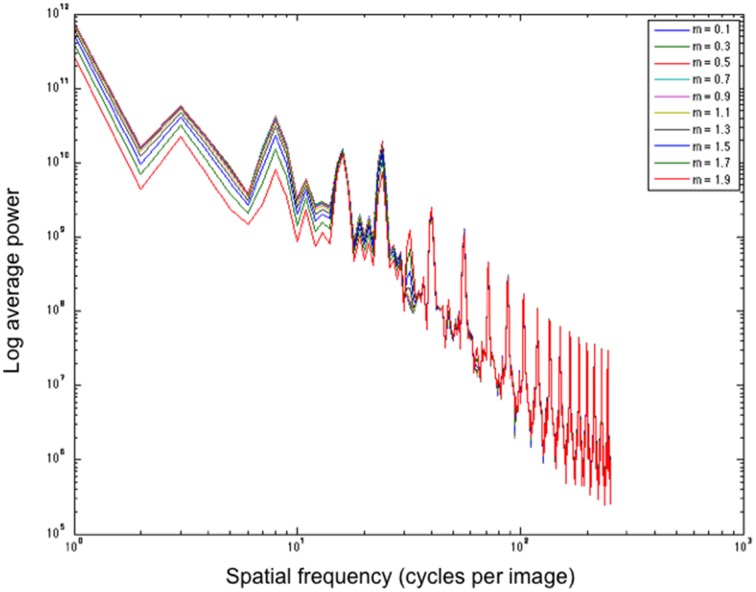
**Average power over spatial frequency of different shape filters applied to White's Illusion (figure a)**. All filters are of size 5 pixels. *m* refers to the exponent.

In a *post-hoc* analysis, we analyse whether filters of a particular shape aid in boosting low SF information, which is postulated by Dakin and Bex (2003) as a driving factor for many illusions. Figure 9 illustrates the effect of different shape filters on the power distribution of a filtered White's Illusion image. Looking at the left side of the graph, we see that different shape filters have an effect on the low SF distributions. Filters with high kurtosis (those that have a low exponent and a sharper distribution) boost low SFs more than filters with low kurtosis (those that have a high exponent and a flatter distribution). The exponential filters therefore provide a mechanism to boost lower SF information more than Gaussian filters.

We emphasize that this study was conducted to investigate filters that are best able to push the power spectra of images toward that of natural images as well as preserve image structure while being resilient to noise. In earlier work, we showed that a filter size selection model helps in extracting and amplifying local image structure (Ghebreab et al., 2009). This model locally selects the smallest filter (extracting high-frequency information) with a response above a noise threshold (ensuring resilience to noise). In a similar fashion, local selection of filter shape may further enhance local image structure. Instead of performing local scale and shape selection in this paper, we study how different types of filters, varying in size and shape, may explain illusions.

The two-stage process of our model uses exponential filters that allow for efficient coding, followed by divisive normalization to boost shallow edges, promoting faithful representation of salient image features. In this way, the filtering stage of our model relies on the Efficient Coding Hypothesis, a theoretical model of sensory coding in the brain (Barlow, 1961). The Efficient Coding Hypothesis states that sensory information is represented in the most efficient way possible, such that it is closely representative of an organism's natural environment. The Efficient Coding Hypothesis is closely related to the Predictive Coding approach (Srinivasan et al., 1982), which states that the representation of sensory information in a statistically efficient way allows sensory systems to reduce redundancies and also provides greater resilience to noise (Barlow, 1961, 2001). In the specific case of our model, there is ample evidence from Basu and Su (2001) that exponential filters are resilient to many types and intensities of noise. From Dakin and Bex (2003) we see that statistical image representation and noise handling complement one another in understanding and predicting lightness illusions. Alongside (Dakin and Bex, 2003), by successfully modeling illusions using properties of image statistics, we support the predictive coding approach proposed by Srinivasan et al. (1982).

In earlier work we showed that globally processing images with filters of different sizes results in scale space image representations that account for different visual phenomena (Ghebreab et al., 2009). We also showed that collapsing scale space representations into a single image representation via local scale selection accounts for even further visual phenomena. This model locally selects the smallest filter (extracting high-frequency information) with a response above a noise threshold (ensuring resilience to noise). In a similar fashion, local selection of filter shape may further enhance local image structure. Instead of performing local scale and shape selection, in this work we first studied if and how different types of filters, varying in size and shape, may explain illusions. We found this is indeed the case. We also tested whether combining different image representations, obtained by globally applying different filters, adds to explaining illusions. The next step in our work would be to determine whether local selection of filter size and shape, based on a model similar to Ghebreab et al. (2009), is able to further explain illusions.

An interesting future direction of study would be to explore additional versions of White's effect, particularly those that have been found to produce an inverted effect (Spehar et al., 1995; Ripamonti and Gerbino, 2001; Spehar et al., 2002). It is well-known that White's effect holds only when the luminance of the two target patches lies between the luminance values of the surrounding gratings (Spehar et al., 1995). Modifying the luminance values of the test patches to double-increments or double-decrements, relative to the gratings, not only drastically reduces the magnitude of illusion, but can also reverse the direction of the illusion from assimilation to contrast (Spehar et al., 1995; Ripamonti and Gerbino, 2001; Spehar et al., 2002). Inverted versions of White's effect have not been successfully accounted for using (Blakeslee and McCourt, 1999)'s ODOG model, according to Spehar et al. (2002). Testing double-increment and double-decrement versions of White's effect in the exponential filter model may further demonstrate its robustness in accounting for an even larger range of lightness illusions.

Another direction for follow-up work would be to investigate the effects of different types and intensities of noise on human perception of lightness illusions and observe how closely these results are matched by our exponential filter model. Dakin and Bex (2003) show that when introducing different levels of noise into their stimuli, their model maintains a close approximation to human performance. However, ODOG has shown discrepancies in matching human response magnitudes for noisy stimuli (Betz et al., 2014). If the exponential filter model demonstrates results similar to human observers in classifying illusory images with noise manipulations, this would provide further support for predictive coding (Srinivasan et al., 1982).

In summary, our study demonstrates that a filter model based on contrast distribution statistics of natural images is able to account for the direction of 21 out of 27 lightness illusions using a single filter. When two filter combinations are considered, the number rises to 23, with asymptotic performance at 24 for an arbitrarily large combination of filter outputs. We observe the effect of incorporating non-linear divisive normalization, providing a better understanding of the role that contrast gain control provides in the perception of these illusions. While short-range normalization only slightly improves the number of correct direction predictions, it considerably reduces the error in predicting illusion magnitude, measured as *RMS_error_*. The exponential filters we employ are not orientation selective, demonstrating that V1-style operations are not required to account for a large number of lightness illusions. Given that these exponential filters could be found as early as the retina, it is possible that the majority of these lightness effects result from pre-cortical operations, leaving only a few to be explained by higher level mechanisms.

## Funding

This work was partially conducted under the Research Priority Program “Brain and Cognition” at the University of Amsterdam and supported by the Dutch national public-private research program COMMIT to SG. This work was also conducted as part of an Endeavour Research Fellowship awarded by the Australian Government to AZ. AZ is supported by the Australian Research Council Centre of Excellence for Cognition and its Disorders (CE110001021) http://www.ccd.edu.au. KB and AZ are supported by the Perception in Action Research Centre (PARC), Macquarie University. Finally, we thank the reviewers for their helpful feedback.

### Conflict of interest statement

The authors declare that the research was conducted in the absence of any commercial or financial relationships that could be construed as a potential conflict of interest.

## Footnotes

^1^In some cases, target patches have equal bordering white and black areas, making it difficult to distinguish whether a contrast or assimilation effect is predominantly present.
